# Efficacy of omadacycline in the treatment of *Legionella pneumonia*: a case report

**DOI:** 10.3389/fcimb.2024.1380312

**Published:** 2024-05-21

**Authors:** Yao Wang, Shui-Min Yi, Si-Min Huang, Wei-Xin Xu, Yi-Wen Wei, Qiang Qu, Jian Qu

**Affiliations:** ^1^ Department of Pharmacy, The Second Xiangya Hospital, Central South University, Institute of Clinical Pharmacy, Central South University, Changsha, China; ^2^ Department of Pharmacy, Traffic Hospital of Hunan Province, Changsha, China; ^3^ Department of Chemistry and Bioengineering, Yichun University, Yichun, China; ^4^ Department of Pharmacy, Foresea Life Insurance Shaoguan Hospital, Shaoguan, China; ^5^ Department of Pharmacy, Xiangya Hospital, Central South University, Changsha, China; ^6^ Hunan Key Laboratory of the Research and Development of Novel Pharmaceutical Preparations, Changsha Medical University, Changsha, China

**Keywords:** omadacycline, *Legionella pneumonia*, *Legionella*, novel tetracycline, moxifloxacin

## Abstract

*Legionella*, one of the main pathogens that causes community-acquired pneumonia, can lead to *Legionella pneumonia*, a condition characterized predominantly by severe pneumonia. This disease, caused by the bacterium *Legionella pneumophila*, can quickly progress to critical pneumonia and is often associated with damage to multiple organs. As a result, it requires close attention in terms of clinical diagnosis and treatment. Omadacycline, a new type of tetracycline derivative belonging to the aminomethylcycline class of antibiotics, is a semi-synthetic compound derived from minocycline. Its key structural feature, the aminomethyl modification, allows omadacycline to overcome bacterial resistance and broadens its range of effectiveness against bacteria. Clinical studies have demonstrated that omadacycline is not metabolized in the body, and patients with hepatic and renal dysfunction do not need to adjust their dosage. This paper reports a case of successful treatment of *Legionella pneumonia* with omadacycline in a patient who initially did not respond to empirical treatment with moxifloxacin. The patient also experienced electrolyte disturbance, as well as dysfunction in the liver and kidneys, delirium, and other related psychiatric symptoms.

## 1 Introduction

*Legionella pneumonia* is a form of pneumonia caused by *Legionella* infection. Predominant symptoms include fever, cough, and shortness of breath, accounting for 2–9% of community-acquired pneumonia cases (Stout and Yu, 1997). *Legionella pneumonia* progresses more rapidly compared to other respiratory atypical pathogen pneumonia, and without timely treatment, patients can develop severe pneumonia, respiratory failure, shock, acute renal failure, and multi-organ dysfunction within a short period of time (Brown, 2004; Dooling et al., 2015; Chahin and Opal, 2017). As many as 44% of patients may need to be transferred to the intensive care unit (ICU), with a case-fatality rate of up to 10%-15% (Brown, 2004; Dooling et al., 2015; Chahin and Opal, 2017). Therefore, early diagnosis and appropriate antibiotic treatment can improve the outcomes for *Legionella* patients. Currently, the preferred therapeutic agents are macrolides and respiratory quinolones (Dennis and Anthony, 2016). However, studies have shown that drug resistance should be considered when quinolones are ineffective in treating *Legionella* (Bruin et al., 2014; Shadoud et al., 2015), and quinolones may cause delirium and other neurological adverse reactions (Stahlmann and Lode, 1999). Omadacycline is a novel tetracycline that has demonstrated anti-*Legionella* activity in both *in vivo* and *in vitro* studies (Dubois, 2020). Its concentration in alveolar macrophages (AM) and alveolar epithelial cell lining fluid (ELF) is higher than that of other novel tetracyclines, making it suitable for treating community-acquired pneumonia (Burgos and Rodvold, 2019). Furthermore, omadacycline does not have the central nervous system adverse effects associated with quinolones (Stahlmann and Lode, 1999). It also has safety advantages for individuals with liver and kidney insufficiency or quinolone intolerance, providing a new treatment option for severe *Legionella pneumonia*. In the reported case, the patient showed a favorable outcome after receiving omadacycline treatment for *Legionella pneumonia*. The patient was emergently sent to the ICU due to their critical condition and was empirically treated with meropenem and moxifloxacin, which are commonly used for severe pneumonia. However, the patient’s condition rapidly deteriorated after the third day of treatment, exhibiting delirium, delusions, agitation, excitement, extubation behavior, and other related psychiatric symptoms, which may be attributed to adverse reactions associated with moxifloxacin (Stahlmann and Lode, 1999). Additionally, the patient experienced acute hepatic and renal insufficiency. Considering the possibility of *Legionella* infection, the treatment was changed to omadacycline, with an initial dose of 200mg followed by a maintenance dose of 100mg once daily intravenously. This treatment showed good efficacy.

## 2 Case description

The patient is a 44-year-old male with a history of hypertension for over three years, currently undergoing treatment with benazepril hydrochloride. His blood pressure control is suboptimal, with self-monitored systolic pressure recorded at 150 mmHg. He also has a history of hyperlipidemia for more than 10 years and regularly takes atorvastatin calcium for lipid reduction. On September 26, 2023, he was admitted to our hospital with symptoms of “recurring fever for five days, worsening with chest discomfort and shortness of breath for over a day.” The patient experienced onset of symptoms in the community. On September 21, 2023, without any apparent precipitating factors, he developed high fever, peaking at 39.7°C, accompanied by chills, sore throat, cough, and expectoration. He was treated at a local hospital, where his blood pressure was measured at 82/59 mmHg, and norepinephrine was administered for blood pressure support. A chest radiograph showed minor inflammation in the lower left lung, but no specific treatment was administered. The patient’s condition continued to fluctuate, leading to his visit to our hospital’s emergency department on September 26.

On the first day of admission, the patient was conscious, with a peak temperature of 40.3°C, pulse rate of 138 beats per minute, respiratory rate of 24 breaths per minute, and blood pressure of 167/81 mmHg. His oxygenation index was 217, and the inflammatory markers were elevated: white blood cell count 12.18×10^9/L, C-reactive protein (CRP) 200 mg/L, and procalcitonin (PCT) 9.17 ng/mL. Liver function tests showed alanine transaminase 255 U/L, aspartate transaminase 79 U/L, albumin 38 g/L, and creatinine (CREA) 467 µmol/L. Initial treatment included 1g of meropenem followed by 0.5g every 8 hours, in combination with 400mg of moxifloxacin daily for anti-infection therapy.

On the third day of admission, patient was still febrile (40°C), blood pressure dropped to 91/49 mmHg, and oxygenation index was 89. He exhibited symptoms of delirium, delusion, agitation, excitement, tube-pulling behavior, suggestive of potential adverse reactions to moxifloxacin. Inflammatory markers showed a significant increase from previous levels: CRP 470 mg/L, PCT 16.25 ng/mL; renal function deteriorated: CREA 850 µmol/L. Continuous renal replacement therapy was initiated. Due to the rapid progression of the disease, the anti-infection regimen was adjusted, discontinuing meropenem and moxifloxacin, and switching to omadacycline (Zhejiang Hisun Pharmaceutical Co., Ltd. No:6221203–11827), starting with a 200-mg dose followed by 100 mg daily, intravenous drip.

**Figure 1:**
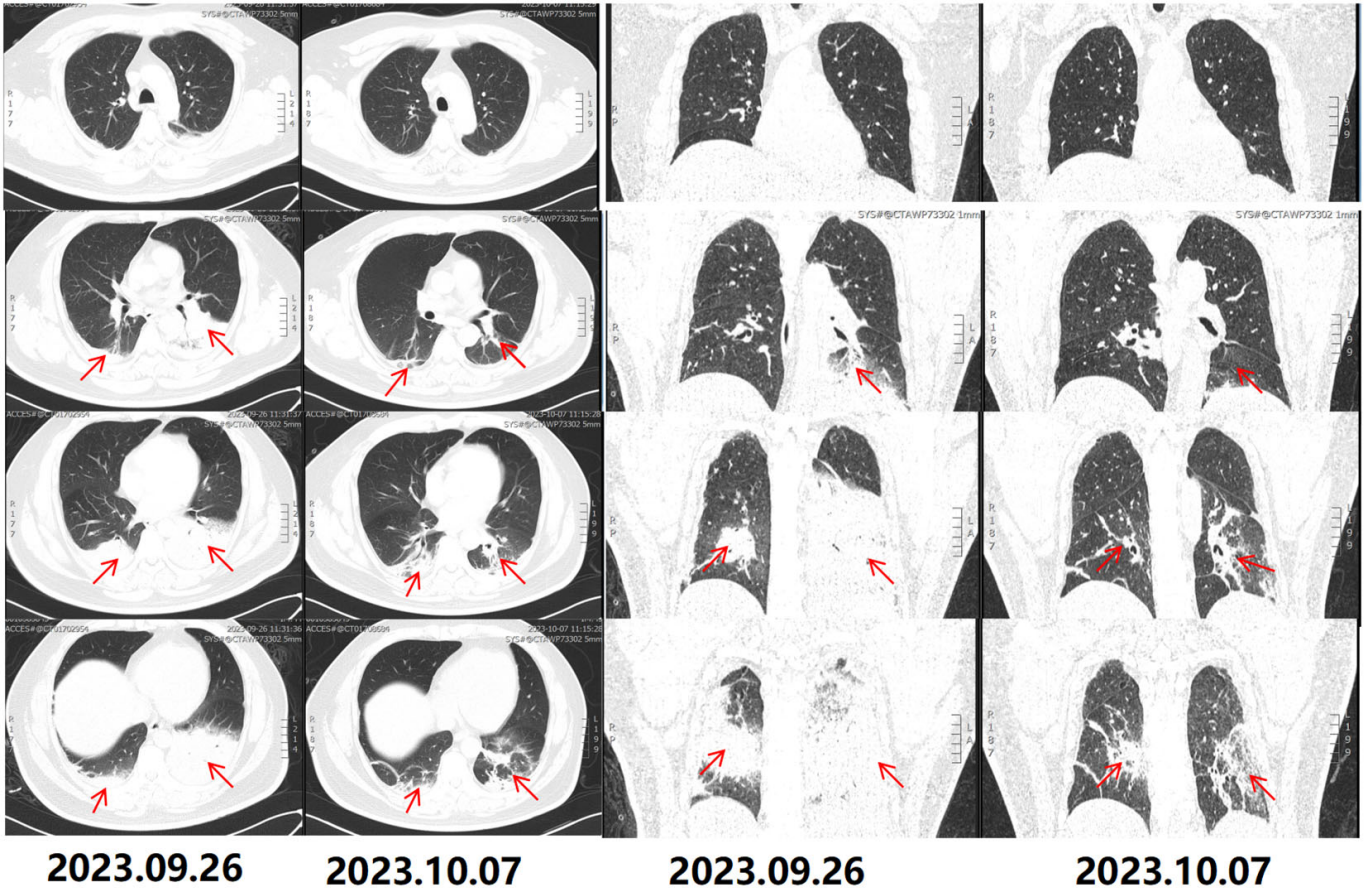
CT scans upon hospital admission. The chest CT on September 26, 2023, reveals bilateral lower lobe consolidation, suggestive of infection, with multiple small and slightly enlarged lymph nodes in the mediastinum. Follow-up lung CT on October 7, 2023, shows partial resolution of the bilateral lower lobe consolidation, indicating improvement of the infection, with the mediastinal lymph nodes unchanged. The red arrows indicate lung consolidation.

On September 29, metagenomic next-generation sequencing of blood samples identified *Legionella pneumophila* with a sequence count of 4 and a coverage of less than 0.01%. No other pathogenic bacteria were found. The mNGS test was done at our hospital’s molecular testing center, cfDNA was extracted from 200 µL plasma using a QIAamp^®^ Circulating Nucleic Acid Kit (Qiagen) following the manufacturer’s protocol. The patient remained on omadacycline treatment for a duration of 10 days. As the oral form of omadacycline was unavailable in China at that time, the injectable preparation was consistently used throughout the treatment course. His body temperature decreased rapidly after the drug was administered, and his inflammatory markers as well as liver and kidney functions gradually improved. Specific data can be seen in **Table 1**. On October 7, a lung CT scan indicated absorption of the consolidation in the lower lobes of both lungs, suggesting an improvement in the infection (**Figure 1**). The patient was transferred to a local hospital for continued treatment on October 10.

**Table 1:** Changes in inflammatory indexes and liver and kidney functions of patients.

Date	Tmax °C (<37.2)	WBC*10^9^/L (3.50- 9.50)	CRP mg/l (0.0- 6.0)	PCT ng/ml (0.000–0.005)	IL6pg/ml (0.0- 7.0)	ALT u/l (9.0- 50.0)	AST u/l (15.0–40.0)	CREA µmol/l (44.0–133.0)
9.26.2023	40.3	12.1	200	9.17	–	255	79	594
9.27.2023	39.2	5.56	–	–	–	103.5	240.4	850
9.28.2023	40	5.11	470	16.25	153.4	97.7	185.9	648
9.29.2023	37.5	6.8	276	10.5	58.1	173.4	302.7	679
9.30.2023	37.3	7.5	–	7.37	43.2	181.6	185.2	870
10.1.2023	37.1	5.45	–	3.19	48.8	146.3	158.1	748
10.3.2023	37.4	–	159	0.707	15.1	111.3	93.9	–
10.6.2023	37.4	6.04	20.25	0.22	3.23	–	–	–
10.9.2023	36.9	8.74	–	–	–	57.1	26.4	221.6

Tmax, Maximum body temperature; WBC, White blood cell count; CRP, C-reactive protein; PCT, Procalcitonin; IL6, Interleukin-6; ALT, Glutamic-pyruvic transaminase; AST, Glutamic oxaloacetic transaminase; CREA, Creatinine.

## 3 Discussion

*Legionella* is a type of bacteria that can cause pneumonia. It is commonly acquired in the community and has an incubation period of 2–14 days. Symptoms of *Legionella pneumonia* include high fever, cough, chills, difficulty breathing, and neurological abnormalities (Cunha, 2016). When treating community-acquired pneumonia, it is important to choose antibiotics that are effective against *Legionella*, such as fluoroquinolones, macrolides, and tetracyclines (Metlay, 2019). For severe cases, a combination of β-lactams and macrolides or respiratory quinolones is recommended (Metlay, 2019). For more severe infections, a shorter treatment course of 7–10 days may be feasible with intravenous/oral azithromycin. However, other antibacterials should be administered for a total duration of 10–21 days (Amsden, 2005). However, studies have shown that *Legionella* can develop resistance to these antibiotics, particularly quinolones (Jonas, 2003). *In vitro* cultures of *Legionella* using quinolones have rapidly induced resistant strains, and mutations in the gyrA region of *Legionella* has been associated with this resistance (Hennebique et al., 2017). This suggests that the empirical use of quinolones may contribute to poor outcomes in severe *Legionella pneumonia*.

Omadacycline tosylate is a novel tetracycline antibiotic that incorporates a lipophilic side group at the C9 position of the D-ring of minocycline. This modification helps overcome the two primary resistance mechanisms of tetracycline, namely efflux pumps and ribosomal protective proteins (LaPlante et al., 2022). It has good activity against various types of bacteria, including Gram-positive, Gram-negative, atypical pathogens, and anaerobes (Editing Group for Multidisciplinary Expert Consensus on the Rational Use of Tetracyclines Commonly Used in Clinical Practice, 2023). It is particularly effective against atypical pathogens like *Mycoplasma*, *Chlamydia* and *Legionella*, making it suitable for treating pneumonia caused by these pathogens (Editing Group for Multidisciplinary Expert Consensus on the Rational Use of Tetracyclines Commonly Used in Clinical Practice, 2023). Omadacycline has a plasma protein binding rate of only about 21%, a high tissue penetration rate, and can be widely distributed throughout the body in most tissues (Lin et al., 2016). Among the new generation tetracyclines, omadacycline has the highest concentration in lung tissue, and the concentration of omadacycline in alveolar macrophages (AM) and alveolar epithelial cell lining fluid (ELF) is higher than that of eravacycline and tigecycline, which makes it suitable for the treatment of lung infections (Gotfried et al., 2017). Common adverse reactions to omadacycline include gastrointestinal symptoms and elevated transaminases (Lin et al., 2023). In this case, the patient did not experience any of these adverse reactions during treatment under our care and observation. Studies have shown that after the treatment with omacycline, there were no statistically significant differences in pharmacokinetics (PK) parameters between healthy subjects and patients with liver or renal insufficiency and the drug was well tolerated, suggesting that no dose adjustment is necessary for the use of omadacycline in patients with hepatic and renal insufficiency (Berg et al., 2018; Kovacs et al., 2020). Studies confirmed that in both the intention-to-treat population and clinically evaluable populations, omadacycline demonstrated non-inferiority in early clinical response rates and investigator-assessed clinical response rates compared to moxifloxacin for the treatment of adult community-acquired bacterial pneumonia (Stets et al., 2019). This suggests that omadacycline is a viable alternative to moxifloxacin, providing more options for clinical treatment of community-acquired bacterial pneumonia.

## 4 Conclusion

*Legionella pneumonia* can rapidly lead to severe and critical illness, causing damage to vital organs. Omadacycline, a new tetracycline with a distinctive aminomethyl structure, has a broader range of antibacterial activity. This report proposes that omadacycline could offer a fresh approach to treating *Legionella pneumonia*, even in patients with liver and kidney dysfunction.

## Data availability statement

The original contributions presented in the study are included in the article/supplementary material. Further inquiries can be directed to the corresponding author.

## Ethics statement

The studies involving humans were approved by ethics committees of the Second Xiangya Hospital of Central South University. The studies were conducted in accordance with the local legislation and institutional requirements. The participants provided their written informed consent to participate in this study. Written informed consent was obtained from the individual(s) for the publication of any potentially identifiable images or data included in this article. Written informed consent was obtained from the participant/patient(s) for the publication of this case report.

## Author contributions

YW: Writing – original draft, Investigation, Writing – review & editing. S-MY: Writing – original draft, Investigation. S-MH: Writing – original draft, Investigation. W-XX: Writing – original draft. Y-WW: Writing – original draft. QQ: Writing – review & editing. JQ: Writing – review & editing, Conceptualization.
